# Dopamine‐Induced Seizure in a Heart Failure Patient: A Case Report and Review of Neurotoxic Implications

**DOI:** 10.1002/ccr3.72302

**Published:** 2026-04-23

**Authors:** Chuanwei Zhao, Yane Yang, Mu Lin, Zeyao Hu, Linhuan Huang

**Affiliations:** ^1^ Department of Cardiology The Second People's Hospital of Baoshan Baoshan Yunnan China; ^2^ Department of Neurology Shantou Central Hospital Shantou Guangdong China

**Keywords:** dopamine, heart failure, neurotoxicity, seizures

## Abstract

In heart failure patients, dopamine therapy, particularly at higher doses, may induce seizures due to its impact on cerebral blood flow and exacerbation of underlying hypoxemia and electrolyte imbalances. Close monitoring and cautious use of dopamine, with consideration of alternative inotropic agents like dobutamine, are essential to mitigate neurotoxic risks.

## 1 Introduction

Dopamine is widely used in the management of heart failure (HF) because of its inotropic properties, which improve cardiac output by enhancing myocardial contractility and stroke volume in a dose‐dependent manner. Specifically, intermediate doses (2–10 μg/kg/min) primarily stimulate β₁‐adrenergic receptors, leading to increased myocardial contractility and cardiac output [1]. While these effects are beneficial at controlled doses, higher doses of dopamine are associated with increased heart rate and peripheral vascular resistance, which may present risks [2]. Despite its therapeutic value, dopamine administration also poses significant challenges, particularly concerning potential neurotoxic effects.

Evidence indicates that dopamine administration can increase cerebral blood flow and metabolic demand, particularly at elevated doses, potentially raising the risk of adverse effects on the central nervous system (CNS) [3, 4]. This risk is particularly relevant in HF patients, who often experience neurological vulnerabilities because of hypoxia and metabolic instability [5, 6]. Dopamine's ability to suppress ventilatory responses could further exacerbate these risks, especially for patients predisposed to seizures [7]. Investigating dopamine's neurotoxic potential in HF patients is therefore crucial to developing safer therapeutic protocols that optimize clinical benefits while minimizing risks. Here, we present a case of dopamine‐induced seizure in a HF patient, underscoring the potential neurological consequences of inotropic therapy.

## 2 Case Presentation/Examination

The patient is a 59‐year‐old male weighing approximately 60 kg, with chronic HF classified as New York Heart Association functional class III, secondary to ischemic cardiomyopathy. His medical history includes type 2 diabetes mellitus diagnosed 10 years earlier, a parietal lobe cerebral infarction approximately 3 years ago without residual neurological deficits, and an anterior myocardial infarction involving the left anterior descending artery about 7 years ago. There was no documented history of hypertension. Apart from the prior stroke, he had no history of epilepsy or other chronic neurological conditions.

He had been taking metformin 500 mg twice daily for glycemic control but had stopped the medication on his own 3 months prior to admission. He had also been on long‐term aspirin monotherapy for secondary cardiovascular prevention, which was discontinued 2 months earlier because of a bleeding duodenal bulb ulcer resulting in hemorrhagic shock. At the time of admission, his regular medications included furosemide 20 mg once daily, spironolactone 20 mg once daily, and trimetazidine 35 mg once daily. He was not taking any antidiabetic or antiplatelet agents. Notably, although the patient was receiving loop and mineralocorticoid receptor diuretics, he was not on other core disease‐modifying therapies recommended by HF guidelines, including angiotensin‐converting enzyme inhibitors, angiotensin receptor blockers, angiotensin receptor–neprilysin inhibitors, beta‐blockers, or sodium–glucose cotransporter two inhibitors. There was no prior documentation of contraindications or intolerance to these medications. He had never been prescribed anticonvulsants and had no recent exposure to medications known to lower seizure threshold, such as systemic corticosteroids, fluoroquinolone or carbapenem antibiotics, or psychotropic drugs.

The patient presented with a 1‐week history of worsening shortness of breath, pronounced orthopnea, and progressive bilateral lower limb edema. On initial examination, his heart rate was 107 beats/min, blood pressure was 85/72 mmHg, respiratory rate was 20 breaths/min, and oxygen saturation was 92% on room air. Electrocardiography demonstrated junctional tachycardia. Cardiac auscultation revealed an S3 gallop, and crackles were heard at the lung bases. Neurologically, he was alert, oriented, and exhibited no focal deficits.

The patient was diagnosed with acute decompensated HF with clinical signs of hypoperfusion. Given his low cardiac output and critically low blood pressure at presentation, dopamine infusion was initiated at a starting dose of 4 μg/kg/min to enhance myocardial contractility and support systemic perfusion. Within the first 24 h, hemodynamic monitoring showed an increase in cardiac index from 1.8 to 2.4 L/min/m^2^, and his blood pressure improved to 107/82 mmHg. His heart rate remained elevated, fluctuating between 100 and 110 beats/min. Repeat electrocardiography continued to show junctional tachycardia, which may have been influenced by underlying autonomic imbalance or the positive inotropic and chronotropic effects of dopamine. The patient received supplemental oxygen at a flow rate of 2 L/min via nasal cannula, but oxygen saturation remained around 92%.

## 3 Methods (Differential Diagnosis, Investigations, and Treatment)

On the second day of dopamine infusion, the patient experienced a generalized tonic seizure lasting approximately 3 min, characterized by sudden loss of consciousness, jaw clenching, and generalized tonic muscle contractions involving all limbs. There was no fixed eye deviation; no involuntary eye movements, limb tremors, or facial twitching; and no frothing at the mouth or tongue biting. Respiration remained stable throughout the episode, which did not progress to status epilepticus, and the patient regained consciousness within approximately 2 min without neurological deficits, residual drowsiness, or headache.

Following the seizure, a neurology consult and a series of evaluations were conducted. Laboratory tests revealed hypokalemia (3.07 mmol/L), hypomagnesemia (0.69 mmol/L), and hypocalcemia (1.78 mmol/L). Compared to admission values, serum potassium had decreased from 3.84 mmol/L to a clearly hypokalemic level, and calcium had declined from 2.08 to 1.78 mmol/L. Serum magnesium was not measured on admission but was found to be significantly reduced after the event. This downward trend in electrolytes, especially in the context of dopamine therapy and diuretic use, suggests a possible contributory role in triggering the seizure. An arterial blood gas analysis indicated persistent hypoxemia with a PaO_2_ of 62 mmHg. Electroencephalography (EEG) showed generalized slowing of brain activity, with no focal epileptiform discharges.

In response to the seizure, dopamine infusion was discontinued. Electrolyte levels were carefully corrected, and the patient's neurological status was closely monitored. Given the possible neurotoxic effects of dopamine and the patient's ongoing hemodynamic instability, dobutamine was initiated as an alternative inotropic agent at a starting dose of 3 μg/kg/min. Over the ensuing 6 h, the cardiac index rose further to 2.8 L/min/m^2^ and mean arterial pressure stabilized at 78 mmHg, while heart rate declined to 92–96 beats/min—consistent with dobutamine's β_1_‐mediated positive inotropy and comparatively milder chronotropic effect. Simultaneously, intravenous potassium, magnesium, and calcium supplementation corrected the initial electrolyte deficits within 12 h. Predischarge laboratory tests confirmed normalization, with serum potassium at 4.51 mmol/L, calcium at 2.14 mmol/L, and magnesium at 0.90 mmol/L.

## 4 Conclusion and Results (Outcome and Follow‐Up)

No additional seizures occurred, and the patient's hemodynamic profile remained stable through 48 h of dobutamine therapy, after which the infusion was weaned without recurrence of neurological events. During follow‐up at 1, 2, and 4 weeks post discharge, the patient reported no recurrent seizures.

This case underscores that dopamine, even at guideline‐recommended dosages, may provoke seizures in patients with HF under conditions of hypoxemia and electrolyte disturbance. Clinicians should closely monitor neurological status and electrolytes in such patients and consider switching to alternative inotropes if neurotoxicity is suspected.

## 5 Discussion

A 59‐year‐old man with low‐output HF developed a tonic seizure while receiving an intermediate‐dose dopamine infusion (4 μg/kg/min). The convulsion occurred against a backdrop of hypoxemia, junctional tachycardia, and newly emergent hypokalemia–hypomagnesaemia, and subsided once dopamine was stopped, electrolytes were corrected, and dobutamine was substituted. The case highlights how even guideline‐level doses of dopamine can precipitate acute neurotoxicity when metabolic and hemodynamic stressors converge.

Dopamine at 2–10 μg/kg/min enhances contractility via β_1_‐adrenergic stimulation and may increase catecholamine tone, with potential adverse effects on cerebral autoregulation in HF circulation. Experimental data suggest that dopaminergic overactivity may lower the seizure threshold through D_2_/D_3_‐linked ion channel modulation [8, 9]. In our patient, concurrent hypoxemia (PaO_2_ 62 mmHg) and electrolyte drift likely amplified this vulnerability; pre‐infusion magnesium was unavailable, and hypomagnesemia was documented after the event. Mechanistically, Mg^2+^ deficiency attenuates the voltage‐dependent Mg^2+^ block of NMDA receptors, shifting cortical networks toward hyperexcitability and seizures [10]. Mg^2+^ depletion also facilitates renal K^+^ wasting through disinhibition of ROMK channels, making concurrent hypokalemia more persistent; together with low‐output hypoxemia, this combined electrolyte and oxygen supply stress may further lower the seizure threshold [11, 12]. Importantly, a magnesium deficit could plausibly have acted as a synergistic cofactor, interacting with dopamine‐related vulnerability rather than being mutually exclusive. The seizure occurred after 24 h of dopamine infusion and resolved promptly after withdrawal, supporting dopamine‐related neurotoxicity, within a susceptible metabolic milieu, as the proximate trigger. Thus, inotropes should be titrated cautiously, and electrolytes monitored closely to prevent neurotoxic complications in decompensated HF settings.

Dopamine was withdrawn and dobutamine 3 μg/kg/min chosen for its β₁‐dominant profile and relatively modest chronotropy, hemodynamics stabilized, and no further seizures occurred. Nevertheless, rare neuromuscular hyperexcitability has been described with dobutamine exposure, including dobutamine‐associated myoclonus and generalized tetany with muscle stiffening and rigidity, particularly in vulnerable patients with advanced HF and renal dysfunction [13, 14, 15]. Contemporary reviews suggest dopexamine, a β_2_/DA agonist with minimal central penetration, can lower afterload and myocardial oxygen demand and may be preferred when cerebral vulnerability is paramount, although supporting data remain limited to small modern series and guideline commentaries [1]. Whichever agent is chosen, early identification and correction of hypoxemia and electrolyte deficits, together with continuous rhythm and oxygenation monitoring, are essential safeguards.

This single‐patient report cannot quantify incidence or establish causality. Continuous EEG and cerebral oximetry were unavailable, so subclinical events might have been missed. Prospective HF registries incorporating neuro‐monitoring and comparing dopamine, dobutamine, and newer agents are needed. Translation of rodent and piglet findings to adult HF should be approached cautiously, yet they remain valuable mechanistic models.

Inotropes can rescue the heart but endanger the brain; vigilant dosing plus rapid correction of hypoxemia and electrolyte drift are critical to prevent dopamine‐linked seizures in HF practice.

## Author Contributions

**Chuanwei Zhao:** conceptualization, funding acquisition, project administration, writing – review and editing. **Yane Yang:** data curation, investigation, writing – original draft. **Mu Lin:** data curation, investigation, writing – original draft. **Zeyao Hu:** data curation, investigation. **Linhuan Huang:** data curation, investigation.

## Funding

This work was supported by the Baoshan Science and Technology Bureau under the Baoshan Science and Technology Planning Project (2024bskjylzd007) and Baoshan University of Traditional Chinese Medicine under the Scientific and Technological Project (2021j005).

## Ethics Statement

The authors have nothing to report.

## Consent

Written informed consent was obtained from the patient for the publication of this case report and any accompanying images.

## Conflicts of Interest

The authors declare no conflicts of interest.

## Data Availability

The data supporting the findings of this case report are not publicly available because of concerns regarding patient privacy and confidentiality. However, de‐identified data may be made available from the corresponding author upon reasonable request and with appropriate institutional or ethics committee approval.
